# Two-Scale Tomography Based Finite Element Modeling of Plasticity and Damage in Aluminum Foams

**DOI:** 10.3390/ma11101984

**Published:** 2018-10-15

**Authors:** Yasin Amani, Sylvain Dancette, Eric Maire, Jérôme Adrien, Joël Lachambre

**Affiliations:** University of Lyon, INSA Lyon, CNRS UMR5510, Laboratoire MATEIS, F-69621 Villeurbanne CEDEX, France; yasin.amani@univ-pau.fr (Y.A.); eric.maire@insa-lyon.fr (E.M.); jerome.adrien@insa-lyon.fr (J.A.); joel.lachambre@insa-lyon.fr (J.L.)

**Keywords:** aluminum foams, intermetallics, X-ray tomography, finite element analysis, damage

## Abstract

In this study, finite element (FE) modeling of open-cell aluminum foams in tension was performed based on laboratory X-ray tomography scans of the materials at two different scales. High-resolution stitching tomography of the initial state allowed local intermetallic particles to be distinguished from internal defects in the solid phase of the foam. Lower-resolution scans were used to monitor the deformation and fracture in situ during loading. 3D image-based FE models of the foams were built to simulate the tensile behavior using a new microstructure-informed Gurson–Tvergaard–Needleman model. The new model allows quantitative consideration of the local presence of brittle intermetallic particles in the prediction of damage. It performs well in the discrimination of potential fracture zones in the foam, and can be easily adapted to any type of architectured material where both the global architecture and local microstructural details should be taken into account in the prediction of damage behavior.

## 1. Introduction

Materials containing gaseous cells are widely found in both nature and engineering applications. Cellular materials can be divided into two different categories based on the continuity of the gaseous phase. The gas phase inside cells can be free (open cell materials) or trapped between cells (closed cell materials). Polymer foams are the most common type of cellular material, but ceramic and metal foams are also produced. Due to their specific structure, cellular materials exhibit a combination of several interesting properties. Mechanically, they hold characteristics like strength, deformability, stiffness, and energy absorption capacity, and are lightweight [1]. Thermally, they are insulators, and some of them are high-temperature-resistant [2]. Acoustically, they are used as effective sound absorbers [3]. In addition, they are frequently applied in other engineering fields, such as packaging, crash-worthiness, and in the production of lightweight sandwich panels [4].

In order to characterize the mechanical properties of cellular materials with complex architecture, an idealized unit cell model assumption was introduced by Ashby [5]. Accordingly, Young’s modulus and plastic collapse strength of the foam are related to an exponential power of the foam’s relative density [1,2,6]. Andrews et al. [7] noticed that the predicted Young’s modulus and strength lie very close to experimental measurements in the case of foams without curvature, corrugation, or internal imperfection.

X-ray micro-computed tomography [8] has been widely used as a non-destructive technique to study yielding mechanism [9] and crack propagation [10]. Finite element (FE) simulations based on tomographic volumes were first used to study the mechanical properties of trabecular bone structures [11]. Maire et al. [12] employed X-ray tomography and morphological granulometry techniques as a generic way to characterize cellular materials to be used for FE calculations. Youssef et al. [13] and Caty et al. [14] developed one of the first methods to build an FE model directly based on the cellular structure obtained by X-ray tomography. Lacroix et al. [15] noted the effect of pore dispersion on the distribution of the FE-computed stress in bone tissue biomaterials. Later, Jeon et al. [16] investigated the deformation and plastic collapse mechanism of closed cell Al foam, and Michailidis et al. [17] determined the stress–strain behavior of open-cell Al and Ni foams. Subsequently, D’Angelo et al. [18] obtained the average Young’s modulus and the stress concentrations within the thinnest sections of SiC ceramic foams. Zhang et al. [19] extended the latter method to explain and predict the rupture of the material based on the contour plot of von Mises stress after simulation. Petit et al. [20] developed a method by running FE simulations and qualitatively defining elastoplastic and damage properties of the aluminum and intermetallics phases. However, no study has yet considered the effect of intermetallics on FE simulation results *quantitatively*.

The present paper focuses on the characteristics of open-cell aluminum foams at two different scales: firstly, the macroscopic cellular structure, and secondly, the local microstructure of the 6101 aluminum alloy constituting the cell walls. The uniaxial tensile modulus and strength of several foams produced by ERG Materials and Aerospace Corp. with different cell sizes are discussed based on X-ray tomography analyses combined with the corresponding image-based FE simulations, where the element behavior is enriched by the local image-based fraction of intermetallics. This new procedure allows the influence of intermetallics on the deformation and damage behavior of the foams to be studied.

## 2. Materials and Experimental Procedures

The studied materials were Duocel^®^ open-cell foams produced and kindly provided by ERG Aerospace Corporation, Oakland, CA, United States. The samples were made of 6101 aluminum alloy, subjected to T6 precipitation-hardening heat treatment. Two foam samples with different cell sizes and testing directions were chosen. Cell sizes of the foam samples were 20 and 30 pores per inch (PPI), corresponding respectively to 0.79 and 1.18 pores per mm. The plasticity and fracture of a 30 PPI foam sample studied in the longitudinal direction were already addressed by Petit [20]. Therefore, the 30 PPI sample was cut in the transverse direction to compare with the study of Petit [20]. The 20 PPI foam sample was cut in the longitudinal direction. The dimensions of the 20 PPI foam sample were 9.4 mm ×6.0 mm × 18.8 mm, and the dimensions of the 30 PPI foam sample were 13.3 mm × 4.8 mm × 10.9 mm.

Table 1 shows the chemical composition of the ERG foam 6101 aluminum alloy characterized by an inductively coupled plasma atomic emission spectrometer by Zhou et al. [21]. Densities of the foam samples were evaluated by weighing on a balance and measuring their dimensions using digital calipers. Afterwards, the relative density of each foam sample was calculated by dividing the global density of the big block of foam by the density of pure Al (2.7g/cm^−3^). Relative densities of 0.0733 and 0.0633 were found for the 20 PPI and 30 PPI foam samples, respectively.

### 2.1. Tomography

Dual-scale laboratory X-ray tomography scanning was used is this study to capture both the macroscopic deformation of the foam during loading and the initial local microstructure, as detailed below. The tomograph (phoenix|x-ray v|tome|x s, GE Company, Boston, MA, USA) produces a series of *N* radiographs corresponding to *N* angular positions of the sample. Based on the Beer–Lambert law, every line integral of the attenuation coefficient along the beam path corresponds to an element in the recorded projection [22]. The resulting images are superimposed information of a three-dimensional (3D) object in a two-dimensional (2D) plane. The detector is a charge-coupled device (Varian Paxscan, Varian Medical Systems Inc., Palo Alto, CA, USA). It records radiographs passed through the sample, which are imported into a commercial reconstruction software (datox|x, GE Company, Boston, MA, USA). The latter uses a filtered back-projection algorithm [23]. The tomograph was operated at 80 kV acceleration voltage using a tungsten transmission target with a 280 μA current. The spot size was between 2 ∼3 μm during all scans, and no filter was used.

First, 3D tomographic images with low resolution (20 μm cubic voxel edge size) were taken to obtain the global structure of the samples. At this resolution, only two phases were imaged: the solid phase and macroscopic void-cells. Each foam sample was then moved toward the X-ray tube to decrease the voxel size so that white intermetallic particles or small cavities could be observed. In the case of the 20 PPI foam sample, the cubic voxel edge size was 4 μm, and it was 6 μm for the 30 PPI foam sample. However, in this high-resolution configuration, the field of view of the detector was not large enough to picture the whole sample. In such cases, the so-called “local tomography” or “region of interest” technique is used. This is a tomography scanning method where portions of the sample are placed in the field of view of the detector during rotation [24].

**Figure 1:**
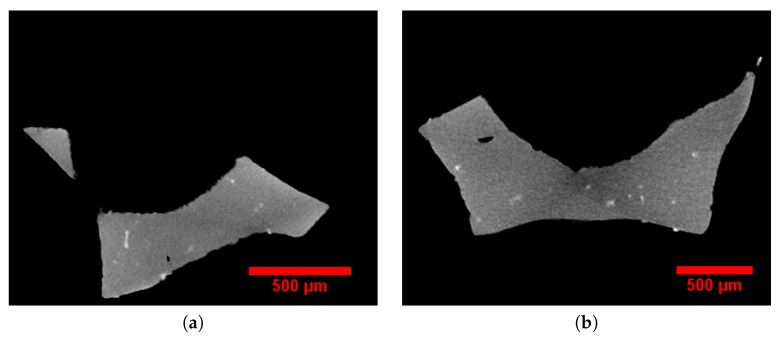
Solid phase defects of foam struts were obtained by local tomography: (**a**) 20 pores per inch (PPI); (**b**) 30 PPI. The light white visible zones are α-AlFeSi (Al_8_Fe_2_Si)- or β-AlFeSi (Al_5_FeSi)-based inclusions.

**Figure 2:**
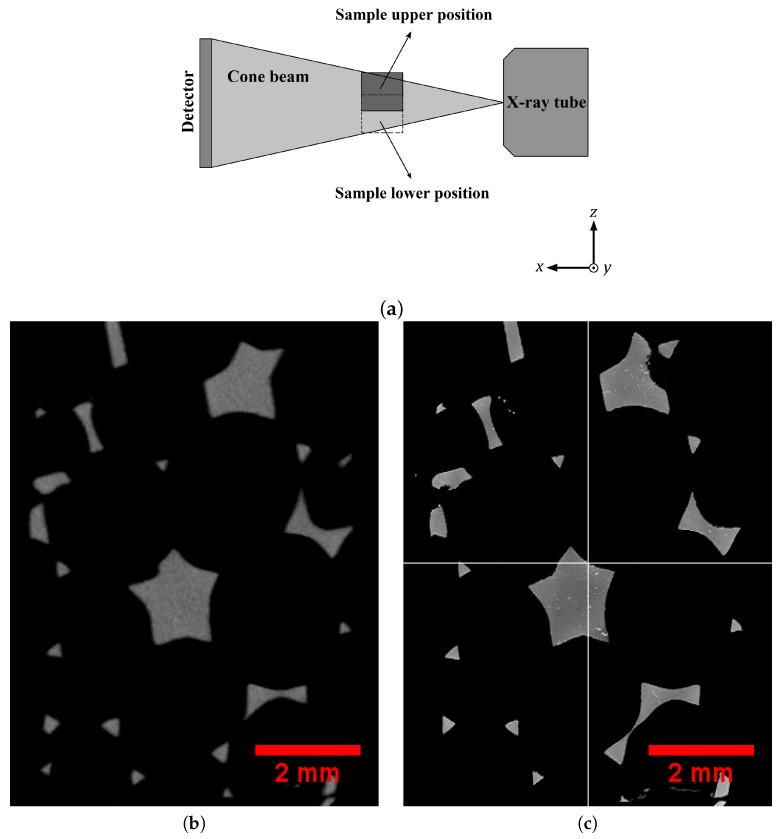
(**a**) Sketch of the stitching tomography setup. Tomography of entire geometry of the 20 PPI sample by: (**b**) global and (**c**) successive local procedure. In (**c**), the intermetallic particles are visible.

The 3D images captured by this technique contained high-resolution details of the solid phase, including intermetallic particles, casting defects, and internal cavities of the foam struts, as shown in Figure 1a,b. Note that gray, white, and black colors correspond to aluminum, intermetallic particles, and void-cells, respectively. These intermetallic particles were mainly α-AlFeSi (Al_8_Fe_2_Si) and β-AlFeSi (Al_5_FeSi) precipitates in the grain boundaries [25]. In order to obtain an entire image of the whole foam sample, the local tomography procedure was repeated many times successively by displacing the center of the sample in a plane parallel to the detector plane. The latter was parallel to the (*y*, *z*) axes of Figure 2a, where a sketch of the setup is illustrated. Then, these high-resolution 3D images were combined and concatenated to retrieve the whole 3D volume, but this time with a small voxel size. The difference between big and small voxel size tomography is illustrated in Figure 2b,c. It is very clear from this image that the higher resolution allows the local presence of intermetallic particles to be captured. Then, aluminum, intermetallic particles, and void-cells were segmented by standard thresholding based on gray levels distribution and were attributed gray (125), white (255), and black (0) 8-bit values for visualization, respectively. The volume fraction of solid (intermetallic particles and aluminum) and gaseous phases were calculated by counting the number of voxels of each corresponding color using the Fiji software [26]. The results are given in Table 2. The amount of porosity in the solid phase was less than 0.01%, which is negligible. The volume fraction of voids and intermetallic particles in the 20 PPI sample were slightly lower than those in the 30 PPI sample. In addition, the image analysis of the tomographic data revealed that most struts presented a close-to-triangular cross section.

The solid phase was analyzed with the local thickness plugin of the Fiji program. The plugin estimates the local thickness by the largest sphere that fits inside the solid phase and contains its voxels. The result of the analysis is a 3D stack of the foam structure, where the local thicknesses can be represented with a given color map.

The average size and distribution of void-cells in three directions were evaluated by the analysis of the gaseous phase of the 3D binary image using a 3D Watershed plugin implemented in the Fiji program. The plugin splits the continuous gaseous phase into non-overlapping void-cells and assigns them different gray levels. These segmented and labeled void-cells do not contain any strut or node. The so-called “Feret” diameter of each segmented cell could be evaluated with another home-made plugin from the minimum and maximum *x*, *y*, and *z* values of its voxels [27].

The average thicknesses of the struts, nodes, and average diameters of void-cells in *x*, *y*, and *z* directions are given in Table 3. The direction in which the foam presents the highest average Feret diameter is called longitudinal direction. The two perpendicular directions are called transverse directions. Consequently, the longitudinal direction for the 20 PPI sample was *z*, and it was *x* for the 30 PPI sample. It can be noted, however, that the geometrical anisotropy of the foams was quite small, which resulted in an almost isotropic mechanical behavior, as documented for example in Ref. [28] on a similar material.

### 2.2. In Situ Tensile Test

In order to investigate the initiation and subsequent propagation of plastic deformation through the foam structures, the two foam samples were glued to steel M3 screws using an epoxy glue (Araldite 2015) prior to being clamped by the steel machine grips [20]. It is noted that the size of the foam samples should be at least eight times larger than the cell size in order to prevent edge effect on measuring Young’s modulus and strength [29]. The samples were then loaded progressively in tension. Tensile tests were performed using an in situ tensile testing machine with a 5 kN load cell. The force transducer (HBM U9B 5kN, Hottinger Baldwin Messtechnik GmbH, Darmstadt, Germany) had a general precision of ±1 N. The loading process was under displacement control at a crosshead displacement speed of 0.001mm/s in order to ensure quasi-static test conditions and proper control of the in situ loading procedure. In accordance with the sample cutting directions detailed above, the 20 PPI foam sample was stretched in the longitudinal direction, while the 30 PPI sample was stretched in the transverse direction. The loading direction corresponds to *z* in Table 3 for both foam samples.

The strain was measured based on the displacement of the top and bottom surfaces of the sample in the 3D tomographic images, obtained for the different deformation steps of the tensile test. Low resolution (20 μm cubic voxel edge size) in situ tomographic scans were performed after every 0.2 mm of crosshead displacement. The (nominal) stress was calculated by dividing the measured force on the load cell by the initial rectangular cross-sectional area of the foam sample.

## 3. Model

### 3.1. Gurson–Tvergaard–Needleman Damage Model

The plastic behavior of the open-cell alloy foam is directly influenced by the properties of the solid phase. The fracture behavior of the solid aluminum phase is governed by ductile damage, namely the nucleation, growth, and coalescence of cavities. Nucleation is mainly due to microcrack initiation (e.g., from pores or defects in the material [30]), second phase particle decohesion, or fracture in the alloy [31]. The growth of cavities occurs by plastic yielding of the matrix surrounding the cavity. Coalescence occurs when neighboring cavities or cracks merge together [32].

In this study, a standard Gurson–Tvergaard–Needleman (GTN) model [33,34,35,36,37] is used to represent the effect of void nucleation and growth in the simulation. The GTN model renders the effect of porosity on the yield locus and its sensitivity to the hydrostatic component of loading. It reduces to a standard isotropic von Mises yielding criterion in the absence of porosity. The governing equation is defined as:

(1) $$\begin{array}{c} \Phi \left(\sigma_{eq},\sigma_{y},\sigma_{H},f\right)={\left(\frac{\sigma_{eq}}{\sigma_{y}}\right)}^{2}+2fq_{1}\cosh\left(\frac{3q_{2}\sigma_{H}}{2\sigma_{y}}\right)-\left(1+q_{3}f^{2}\right)=0, \end{array}$$

where Φ is the yield function, $q_{1}$, $q_{2}$, and $q_{3}$ are calibrating parameters, $\sigma_{eq}$ is the von Mises equivalent stress, $\sigma_{y}$ is the yield stress, $\sigma_{H}$ is the hydrostatic stress, and *f* is the void volume fraction (VVF) in the matrix. In addition, starting from an initial void volume fraction $f_{0}$, the total change in *f*, noted $\dot{f}$, is defined as [34]:

(2) $$\begin{array}{c} \dot{f}={\dot{f}}_{gr}+{\dot{f}}_{nucl}=\left(1-f\right)\;tr\left({\dot{\varepsilon }}^{pl}\right)+\frac{f_{N}}{s_{N}\sqrt{2\pi }}\exp\left[-\frac{1}{2}{\left(\frac{\varepsilon_{eq}^{pl}-\varepsilon_{N}}{s_{N}}\right)}^{2}\right]{\dot{\varepsilon }}_{eq}^{pl}, \end{array}$$

where ${\dot{f}}_{nucl}$ is the contribution of nucleating voids; ${\dot{f}}_{gr}$ is the void growth rate, which is based on mass conservation and directly proportional to the hydrostatic component of plastic strain rate tensor $tr({\dot{\varepsilon }}^{pl})$; and $\varepsilon_{eq}^{pl}$ is the equivalent plastic strain. $\varepsilon_{N}$ and $s_{N}$ are the mean value and standard deviation of the normal nucleation distribution. $f_{N}$ is the volume fraction of the nucleated voids. The power law work hardening used in this study is defined as [38]:

(3) $$\begin{array}{c} \frac{\sigma_{y}}{\sigma_{0}}={\left(\frac{\sigma_{y}}{\sigma_{0}}+\frac{3G}{\sigma_{0}}\varepsilon^{pl}\right)}^{N}, \end{array}$$

where $\sigma_{0}$ is the initial yield stress, *N* is the hardening exponent, *G* the elastic shear modulus and $\varepsilon^{pl}$ is the plastic strain. In addition, the initiation of necking in tension takes place according to a Considère criterion when the work hardening rate converges to the current yield stress:

(4) $$\begin{array}{c} \frac{\partial \sigma_{y}}{\partial \varepsilon^{pl}}=\sigma_{y}. \end{array}$$

### 3.2. Mesh Generation

In this study, the initial 3D tomographic volumes of the foam samples were used to generate 3D image-based FE meshes. First, a surface mesh was generated from the solid phase boundaries in the volume. Next, the surface mesh was simplified and remeshed to reduce the number of triangles while preserving a proper description of the surface. Finally, the solid volume was filled by first-order tetrahedra [13] (C3D4 elements in the Abaqus software), allowing for explicit non-linear simulations including damage and fracture. The whole meshing procedure was performed using the commercial Avizo^®^ software [39]. Each foam sample was meshed with four different mesh sizes in order to investigate the mesh size sensitivity of the results, as detailed in Section 3.4. The number of tetrahedra and nodes of the reference volume meshes are detailed in Table 4.

Two classes of FE models were considered in this work: (i) *homogeneous* (or microstructure “blind”) models, where the local constitutive behavior corresponds to the nominal average behavior of the aluminum alloy everywhere; and (ii) *heterogeneous* models, where the local constitutive behavior depends on the local microstructure (intermetallic fraction in the present case) as informed from the detailed initial 3D tomographic scans of the samples.

**Figure 3:**
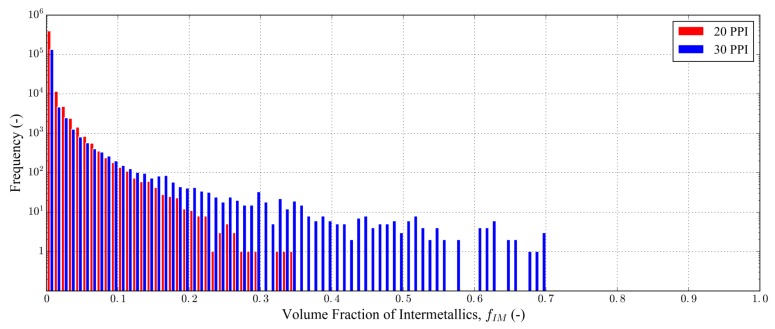
Distributions of intermetallic particles volume fraction in the finite element (FE) tetrahedra for the 20 PPI and 30 PPI foam samples.

The procedure used to generate the *heterogeneous* FE model is described in [40] and briefly recalled here. For each element of the 3D image-based FE mesh, a python script retrieves the voxels of the segmented tomographic volume located in the element interior. The number of white (intermetallic) voxels is then evaluated in order to compute the local intermetallic volume fraction $f_{IM}$ in the element. As a result, the tetrahedra of the volume mesh are tagged depending on the volume fraction of intermetallic particles regrouped into 100 equidistant classes with $f_{IM}$ varying from 0.0 to 1.0. The distributions of the intermetallic volume fraction in the tetrahedra are illustrated in Figure 3 for the 20 PPI and 30 PPI foam samples.

### 3.3. Identification of the Constitutive Model Parameters

The identification of constitutive model parameters is a challenging task, especially when it comes to the local behavior of the solid (metallic) phase in macroporous structures like the present foam samples. Several studies demonstrated that the tensile mechanical behavior of individual struts extracted from the foam might be significantly different from the behavior of the corresponding bulk metal [41,42,43], even subjected to the same hardening heat treatment. The problem becomes even more complicated if one would like to take the local microstructure (e.g., local intermetallic fraction here) of foam struts into account in the constitutive modeling. The present work proposes to rely on axisymmetric FE unit cell calculation to compute the local constitutive behavior of the different aluminum matrix composites with various intermetallic volume fractions that are available in each tetrahedron of foam sample mesh.

**Figure 4:**
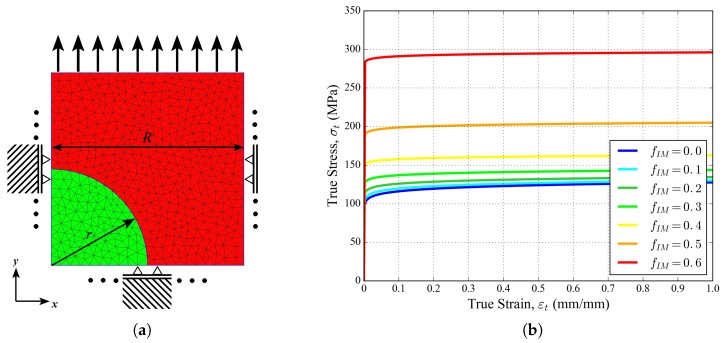
(**a**) 2D axisymmetric model of the aluminum matrix material (red) with a given intermetallic (green) volume fraction $f_{IM}$. The right exterior line remains straight and vertical during loading, but is free to move in the radial direction. (**b**) Resulting hardening behavior for increasing intermetallic volume fraction.

The 2D axisymmetric cell model is illustrated in Figure 4a. Varying the intermetallic (green) radius *r* allows to cover intermetallic volume fractions $f_{IM}$ from 0.0 to 0.6 in order to compute the resulting composite behavior to be considered for the corresponding elements in the distribution of Figure 3. A linear elastic behavior with a Young’s modulus of E= 160 GPa and a Poisson’s ratio ν= 0.33 was considered for the intermetallic properties [20]. The power-law hardening of Equation (3) was used for the aluminum phase, with E= 70 GPa, ν= 0.33, an initial yield stress $\sigma_{0}=$ 97 MPa, and a hardening exponent N= 0.052. This initial yield stress corresponds to the lower range of individual strut yield stresses measured by [41] on a similar foam. The resulting tensile stress–strain curves obtained for increasing intermetallic volume fraction $f_{IM}$ are illustrated in Figure 4b. They were extracted along with the corresponding Young’s moduli (ranging from 70 GPa for pure aluminum to 115 GPa for $f_{IM}=$ 0.6) to define the elasto-plastic properties of the foam tetrahedra in the different classes of Figure 3.

**Figure 5:**
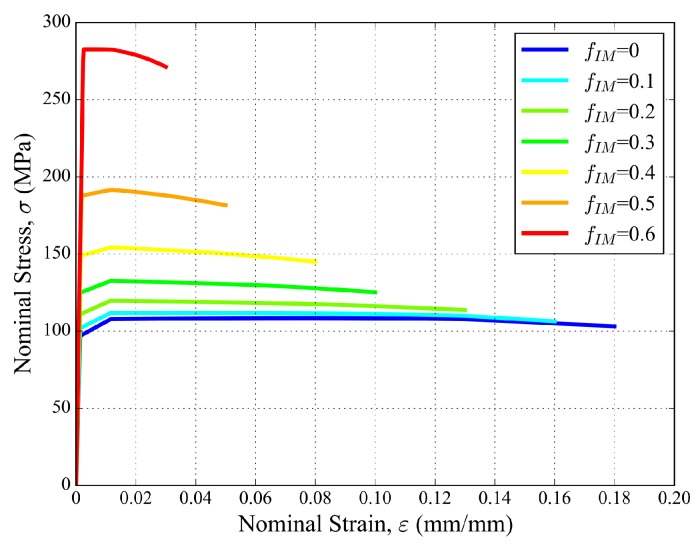
Tensile stress–strain curves of aluminum matrix materials including GTN damage, for increasing fraction of intermetallic particles.

Damage model parameters were also identified for each element class (each value of $f_{IM}$). Following [36], the calibrating parameters of the GTN yield function in Equation (1) were chosen as $q_{1}=1.5$, $q_{2}=1$, and $q_{3}=2.25$. In the nucleation part of Equation (2), the volume fraction of nucleating voids was taken as $f_{N}=$ 0.04 [20,44]. The initial void volume fraction $f_{0}$ was taken as zero, which is consistent with the negligible initial porosity of the solid phase measured in the tomographic volumes (Section 2.1). $\varepsilon_{N}$ and $s_{N}$ were gradually decreased with the increasing value of $f_{IM}$ to mimic the transition towards a brittle behavior with the increase in intermetallic fraction in the material (Table 5). Figure 5 illustrates the resulting tensile stress–strain behaviors, including GTN damage of the corresponding aluminum matrix materials. The increase in volume fraction of intermetallic particles clearly results in a stiffer, stronger, but more brittle behavior. This will impact the constitutive response of the tetrahedra with a high intermetallic fraction in the foam FE mesh.

### 3.4. Simulation Conditions

The FE approach described in the present study consists of a uniaxial monotonous tensile loading applied to the 3D image-based FE meshes generated in the previous section. Two nodes, one on the top surface and another on the bottom surface of the foam samples, were assigned as master nodes. *z* displacement degree of freedom of each node on the top surface was constrained to be the same as the one of the top master node, which was assigned a positive displacement $u_{z}$. Similarly, the *z* displacement degree of freedom of each node on the bottom surface was constrained to be the same as the one of the bottom master node, which was blocked. Furthermore, nodes located on two central lines on the top and bottom faces were constrained by imposing $u_{x}$ = 0 to prevent the potential rotation of the sample about the tensile axis, which is inhibited experimentally by the machine grips.

**Figure 6:**
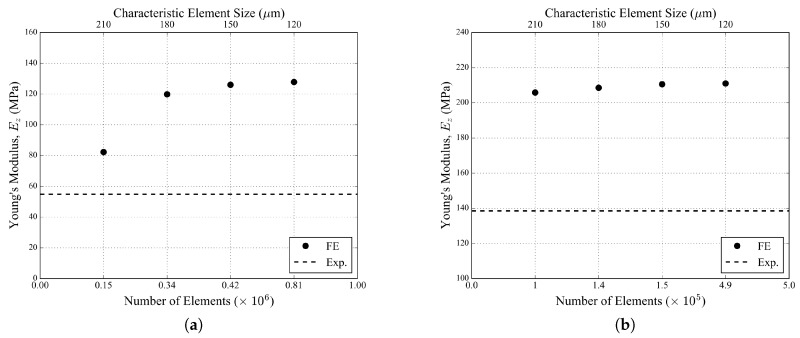
Apparent Young’s modulus convergence tests for (**a**) 20 PPI and (**b**) 30 PPI foam samples in the case of the homogeneous non-porous models.

The sensitivity of the simulation results with respect to the element size is illustrated in Figure 6a,b with the predicted apparent Young’s modulus obtained with the homogeneous non-porous models. It converged to about 125 MPa with decreasing element size in the case of the 20 PPI foam sample and to about 210 MPa in the case of the 30 PPI foam sample. The characteristic element size of 150 μm was therefore chosen as the reference element size for both samples in Table 4. However, one can note that these predicted values of Young’s moduli were overestimated with respect to the experimental measures based on the (3D) image-based macroscopic tensile strain. Two modeling choices in the present study might contribute to this situation: (i) The use of first-order tetrahedral elements imposed by the complexity of the structure and the compatibility with the Explicit solver of the Abaqus FE software. First-order tetrahedra are known to be too stiff in bending-dominated problems [38]. (ii) The uniaxial tension boundary conditions applied in the simulations slightly differ from the effective boundary conditions in the experiments.

## 4. Results and Discussion

**Figure 7:**
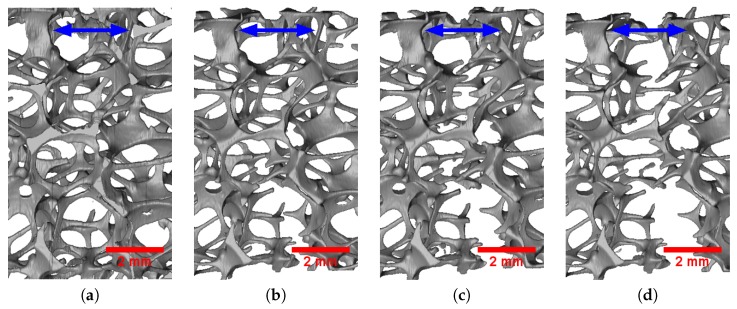
Deformation of the 20 PPI foam sample during the in situ tensile test at nominal strain: (**a**) 0.000; (**b**) 0.019; (**c**) 0.040; (**d**) 0.064. The blue arrow indicates the loading direction.

**Figure 8:**
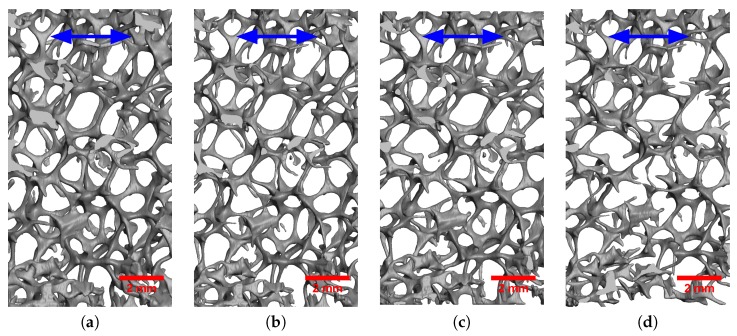
Deformation of the 30 PPI foam sample during the in situ tensile test at nominal strain: (**a**) 0.000; (**b**) 0.031; (**c**) 0.085; (**d**) 0.142. The blue arrow indicates the loading direction.

Figure 7 and Figure 8 respectively illustrate the progressive deformation of the 20 PPI and 30 PPI foam samples during the in situ tensile tests. Spatially uniform straining was observed until the first fracture happened in a strut. Both plastic stretching and plastic bending of the struts might be observed during the tensile deformation of foam blocks. Struts approximately parallel to the loading direction were mostly stretched, while the others were deformed first by bending and then potentially by stretching once they were aligned with the tensile direction. It was observed here that the majority of the struts were not parallel to the loading axis. Therefore, the deformation of the foam blocks was dominated by bending for small levels of elongation. The deformation then shifted to a stretching-dominated mode after significant elongation and re-alignment of the struts. It should be noted that bending-dominated deformation tended to lower the apparent stiffness and strength of the foam samples, while stretching dominated deformation rather tended to increase them.

Fracture in the foam blocks initiated in struts that were parallel to the loading direction and stretched. Subsequent propagation of fracture was achieved in the neighboring struts, mostly reclined with respect to the loading direction and exhibiting first bending and then stretching deformation prior to collapse. Once some struts broke, the fractured area expanded to the adjacent cells.

**Figure 9:**
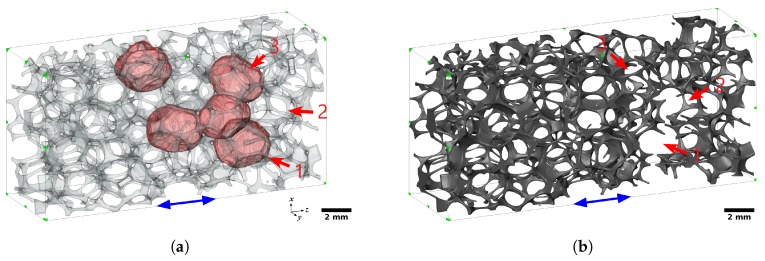
(**a**) The five biggest void-cells and (**b**) the final fractured state of the 20 PPI foam sample. The blue arrow indicates the loading direction. Cells of interest are labeled 1 to 3.

**Figure 10:**
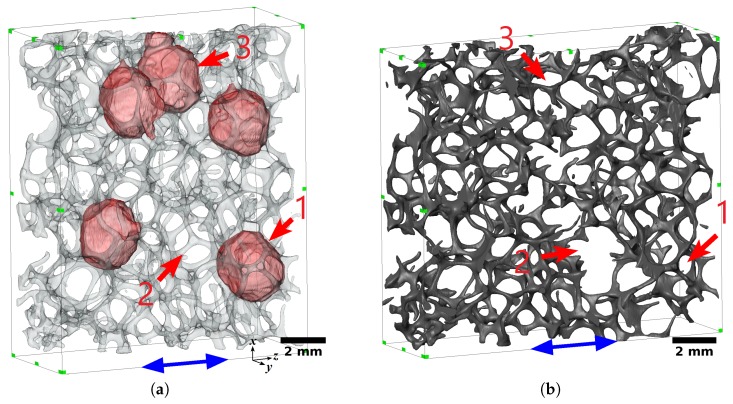
(**a**) The five biggest void-cells and (**b**) the final fractured state of the 30 PPI foam sample. The blue arrow indicates the loading direction. Cells of interest are labeled 1 to 3.

Figure 9a illustrates the five biggest void-cells of the 20 PPI foam sample, highlighted in red. Figure 9b shows the final step of the fractured foam sample during the in situ tensile test. The fracture area was located in the vicinity of the three big neighboring cells, and passed through two of them (numbers 1 and 3, Figure 9b). In the case of the 30 PPI foam sample, the five biggest cells (Figure 10a) were more scattered than those of the 20 PPI sample. The fracture area was located between the five biggest cells and ended into two of them (numbers 1 and 3, Figure 10b). Furthermore, the 30 PPI foam sample, which was elongated in the transversal direction, was contracted significantly in the *x* and *y* directions.

In order to study the effect of intermetallic particles in the solid phase, four FE simulations were performed for each foam sample: (i) *homogeneous* non-porous J2 model, (ii) *heterogeneous* non-porous J2 model, (iii) *homogeneous* porous GTN model, and (iv) *heterogeneous* porous GTN model. J2 models here correspond to standard isotropic (von Mises) plasticity.

**Figure 11:**
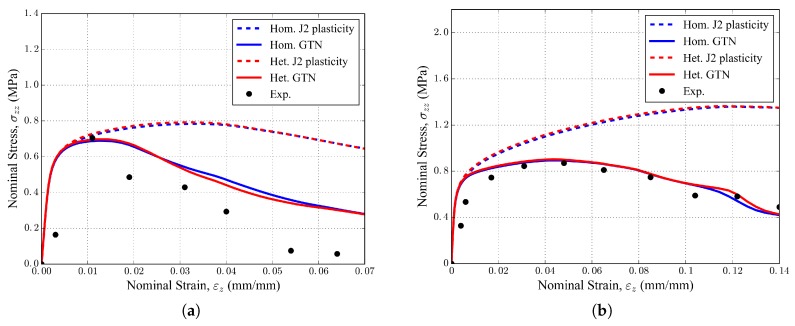
Macroscopic experimental and FE tensile curves of the (**a**) 20 PPI and (**b**) 30 PPI foam samples.

Figure 11a,b illustrate the macroscopic tensile curves obtained with the 20 PPI and 30 PPI foam samples in the experiments and simulations. The black points correspond to experimental measurements (note that only the first point laid in the linear elastic regime for each foam sample). The dashed lines correspond to the simulations with isotropic non-porous plasticity (J2 plasticity). The bold lines correspond to the simulations with the GTN damage model. The blue curves correspond to the *homogeneous* simulations with an aluminum matrix without intermetallic particles included. The red curves correspond to the *heterogeneous* simulations where the local presence of intermetallic particles with a given volume fraction $f_{IM}$ is taken into account in the constitutive behavior of the elements. It is clear that taking the presence of intermetallic particles into account in the FE simulations *did not* affect the calculated macroscopic stress–strain curves significantly. However, the non-porous models did not capture the last stages of foam deformation properly, overestimating the reaction stress in the absence of damage.

The tensile curves of Figure 11 exhibited some macroscopic hardening behavior and a peak stress attained after a few percents of macroscopic deformation. The hardening arose from both the geometrical rearrangement of the struts during loading and the constitutive strain hardening of the aluminum solid phase. The 20 PPI foam sample exhibited a 55 MPa Young’s modulus and 0.70 MPa tensile strength. The 30 PPI foam sample exhibited a 138Pa Young’s modulus and 0.87 MPa tensile strength, which was both stiffer and stronger than the 20 PPI sample due to the smaller size of the cells. One can note also the higher apparent ductility of the 30 PPI foam sample, accepting a larger level of macroscopic deformation before collapse.

**Figure 12:**
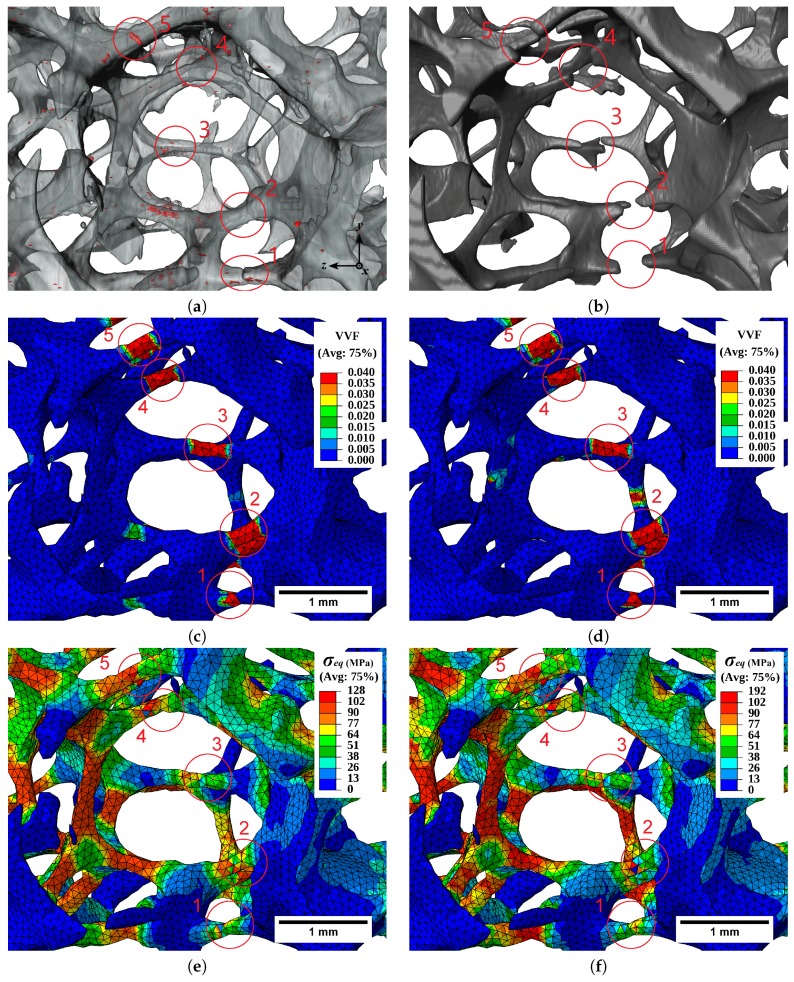
(**a**) Distribution of intermetallic particles in the solid phase of the 20 PPI sample. (**b**) Fractured struts of the foam. FE contour plots (at 6.4% strain) of VVF with the (**c**) homogeneous and (**d**) heterogeneous GTN models. Corresponding contour plots of equivalent stress with the (**e**) homogeneous and (**f**) heterogeneous GTN models. Struts of interest are labeled 1 to 5.

Figure 12a,b illustrate the presence of intermetallic particles in a subregion of the 20 PPI foam sample, before the test and after fracture of the struts. It shows that strut numbers 1 and 5 broke in the vicinity of clusters of intermetallic particles. This was also the case of strut number 4, yet with a smaller amount of particles. On the contrary, other regions with important clusters of intermetallic particles did not fail, illustrating the complexity of the situation leading to fracture. Several factors contributed and competed to trigger fracture, including the loading mode (or misalignment of the struts with the tensile load), local geometrical stress concentration (e.g., due to local reduction in cross section), and the presence of hard and brittle intermetallic particles.

The contour plots of void volume fraction computed in the FE simulations with the homogeneous and the heterogeneous GTN models are illustrated in Figure 12c,d. Figure 12d with the heterogeneous model shows, for example, a similar VVF to the homogeneous model (Figure 12c) in strut number 5 at the place of fracture, despite the presence of a cluster of particles that was not taken into account in the homogeneous model. Strut number 2 illustrates that similar levels of VVF could also be reached in the absence of particles due to geometrical plastic strain localization, which was predicted in both homogeneous and heterogeneous models. Strut number 1 illustrates that such localization of plastic flow due to local cross section reduction can be the critical point even in the presence of particles, in which case both models again provided comparable local VVF.

Similar comparisons can be made in terms of equivalent stress distribution in the struts in Figure 12e,f). Stress concentration arose either from local reduction of cross section, in which case both models provided comparable results, or with an additional contribution of the local presence of hard particles. One can note, however, that the sole prediction of stress concentration would fail in systematically discriminating the potential fracture zones at the microscopic level, justifying the use of the GTN porous plasticity model in the present work.

This study illustrates that stress analysis in metal foams is not straightforward due to the non-uniform stress distribution arising from the non-uniform geometry [45] and microstructure. The distribution of von Mises stress in the foam under loading depends on size, orientation, and spatial arrangement of the cells in a complex manner [17], in addition to the local presence of hard particles. It was however observed here that tensile loading of the foams promoted stress concentration in the struts of the biggest void-cells. The subsequent fracture of these struts formed the fracture plane.

Other studies have also suggested that the fracture mode of ERG foam struts depends on the type of precipitates in the Al matrix. The failure of the struts was observed to begin by realignment and ductile transgranular fracture of struts in the fracture plane [25]. The fracture mode later shifted to major brittle intergranular and minor ductile transgranular failure of the remaining struts due to the presence of α-AlFeSi (Al_8_Fe_2_Si) and β-AlFeSi (Al_5_FeSi) precipitates in the grain boundaries. The experimental protocol of the present study using laboratory tomography does not allow us to easily distinguish these different types of precipitates. The use of in situ synchrotron tomography might be an interesting perspective in this context.

As concerns modeling, current limits of the present approach might be overcome in the future by: (i) using a modified FE simulation environment allowing to alleviate the requirement for first-order tetrahedral elements in highly non-linear simulations with extensive damage development, (ii) using more realistic boundary conditions (e.g., directly mapped from the 3D in situ images), (iii) better identifying the local strut constitutive behavior. Indeed, in this study and in the recent one of Petit et al. [20], direct measures of yield stresses and hardening behavior on single-strut micro-tensile tests [41,43] had to be adjusted to properly reproduce the macroscopic foam response. A perspective might consist of taking the crystallographic orientation of the grains in the struts into account for the calculation of their plastic behavior. This could have significant consequences in this material, since some struts are made of a limited number of grains [21], and could exhibit rather anisotropic plastic behavior. Image-based crystal plasticity FE simulations [46] might be particularly interesting in this context.

As a final comment, the present study shows that *quantitatively* taking the local intermetallic particles into account in the prediction of the foam mechanical behavior does not really affect the macroscopic response, but allows a rather good discrimination of the critical zones for fracture in the structure. This confirms the *qualitative* conclusions and prospects of Petit et al. [20]. Nevertheless, it also tends to indicate in this particular case that accounting for intermetallics in such a homogenized GTN damage simulation framework is only of secondary importance for the prediction of fracture, after the proper accounting for local geometry and loading mode of the nodes/struts. Different conclusions were recently found in the study of Amani et al., [40] where the presence of local heterogeneous microporosity in additively manufactured lattice structures had more significant consequences on the prediction of fracture behavior, yet with a higher global volume fraction and a different type of defect (process-induced cavities) as compared to the present study. Note also that direct full-field modeling of the small intermetallics in the struts would allow a more accurate prediction of local stress heterogeneity and fracture onset. However, such full-field simulations would require a severe increase in computational resources and would hardly be applicable on real-size samples. Moreover, the identification of the local constitutive behavior in the struts remains a challenge. The micromechanical approach developed in this study is based on cell-calculation and the GTN model to derive the constitutive behavior of local particle-rich regions of the foam. It can constitute the basis for a wider range of applications to complement 3D image-based FE studies of macroporous materials, which are now well established. This might include, for example, any multiscale investigation of architectured materials where both (i) a global description of the macroscopic structure, but also (ii) local image-based microstructural details are expected to be required to understand and predict the macroscopic behavior of the material.

## 5. Conclusions

A method was developed in this study to investigate the tensile behavior of an ERG foam provided in two cell sizes, by taking advantage of in situ tensile tests under microtomography and microstructure-informed FE modeling:

- The internal architecture of the solid phase of the foam was analyzed using high-resolution local tomography, providing elaborate quantitative data of the location of internal defects (e.g., internal microvoids and intermetallic particles inside the sample).
- The deformation and fracture mechanisms of the foam were studied in situ in tension using lower-resolution scans.
- Image-based FE simulation of the tests was performed using a microstructure-informed porous plasticity (Gurson–Tvergaard–Needleman, GTN) model, quantitatively taking the local presence of brittle intermetallic particles into account (the so-called *heterogeneous* GTN model).
- The *heterogeneous* model performed well in the discrimination of potential fracture zones, but did not perform better than the corresponding *homogeneous* (or microstructure “blind”) model in the prediction of global stress–strain curves.
- The procedure can be easily utilized for the investigation of other types of architectured materials where both the macroscopic architecture and local microstructural details are expected to be required in order to understand and predict the material behavior.

## Figures and Tables

**Table 1:** Chemical composition of the 6101 aluminum foam in weight percent (wt.%). Data from Zhou et al. [21].

Element	Cu	Mg	Mn	Si	Fe	Zn	B
Content	0.03	0.19	0.01	0.27	0.12	0.01	0.03

**Table 2:** Volume fractions in percent (%) of the different phases in the foam samples.

Cell Size (PPI)	Void	Aluminium	Intermetallic Particles
20	92.67	7.31	0.02
30	93.07	6.89	0.04

**Table 3:** Geometric characteristics of the studied foams.

Cell Size (PPI)	20	30
Strut thickness (mm)	0.16 ± 0.04	0.16 ± 0.04
Node thickness (mm)	0.38 ± 0.10	0.36 ± 0.04
Void-cell dimension in *x* (mm)	2.60 ± 0.20	2.57 ± 0.49
Void-cell dimension in *y* (mm)	2.84 ± 0.23	2.36 ± 0.49
Void-cell dimension in *z* (mm)	3.04 ± 0.55	2.47 ± 0.32

**Table 4:** Characteristics of the reference volume meshes for the two foam samples.

Cell Size (PPI)	Number of Nodes	Number of Elements	Characteristic Element Size (microns)
20	124,267	419,846	150
30	50,611	146,660	150

**Table 5:** Parameters of the Gurson–Tvergaard–Needleman (GTN) nucleation model for increasing volume fraction of intermetallic particles.

Intermetallic Fraction $f_{\mathrm{IM}}$			
	$\varepsilon_{N}$	$s_{N}$	$f_{N}$
0.0	0.18	0.06	0.04
0.1	0.16	0.05	0.04
0.2	0.13	0.04	0.04
0.3	0.10	0.03	0.04
0.4	0.08	0.03	0.04
0.5	0.05	0.02	0.04
0.6	0.03	0.01	0.04

## Abbreviations

The following abbreviations are used in this manuscript:

FE	Finite Element
GTN	Gurson–Tvergaard–Needleman
PPI	Pores Per Inch
VVF	Void Volume Fraction
